# Screening and Comparison of Lignin Degradation Microbial Consortia from Wooden Antiques

**DOI:** 10.3390/molecules26102862

**Published:** 2021-05-12

**Authors:** Wen Zhang, Xueyan Ren, Qiong Lei, Lei Wang

**Affiliations:** 1 School of Biology and Environmental Engineering, Zhejiang Shuren University, Hangzhou 310015, China; 2 School of Engineering, Westlake University, Hangzhou 310024, China; renxueyan@westlake.edu.cn; 3 Institute of Advanced Technology, Westlake Institute for Advanced Study, Hangzhou 310024, China; 4 Jingzhou Conservation Center of Hubei Province, Wood Lacquer Protection Research Department, Jingzhou 434020, China; leiqiong99@163.com

**Keywords:** lignin degradation, microbial consortium, biomass, synergetic degradation

## Abstract

Lignin, which is a component of wood, is difficult to degrade in nature. However, serious decay caused by microbial consortia can happen to wooden antiques during the preservation process. This study successfully screened four microbial consortia with lignin degradation capabilities (J-1, J-6, J-8 and J-15) from decayed wooden antiques. Their compositions were identified by genomic sequencing, while the degradation products were analyzed by GC-MS. The lignin degradation efficiency of J-6 reached 54% after 48 h with an initial lignin concentration of 0.5 g/L at pH 4 and rotation speed of 200 rpm. The fungal consortium of J-6 contained Saccharomycetales (98.92%) and Ascomycota (0.56%), which accounted for 31% of the total biomass. The main bacteria in J-6 were *Shinella* sp. (47.38%), *Cupriavidus* sp. (29.84%), and *Bosea* sp. (7.96%). The strongest degradation performance of J-6 corresponded to its composition, where Saccharomycetales likely adapted to the system and improved lignin degradation enzymes activities, and the abundant bacterial consortium accelerated lignin decomposition. Our work demonstrated the potential utilization of microbial consortia via the synergy of microbial consortia, which may overcome the shortcomings of traditional lignin biodegradation when using a single strain, and the potential use of J-6 for lignin degradation/removal applications.

## 1. Introduction

Lignin, which is an aromatic heteropolymer that accounts for 15–30% of lignocellulosic biomass, is the most abundant source of renewable aromatic carbon in nature [1]. Lignin consists of phenylpropane units combined with different types of linkages, including β-O-4, α-O-4, 4-O-5, β-β, β-5, 5-5, and β-1 [2]. It is a recalcitrant compound due to its high molecular weight, structural complexity, and relative insolubility. It can result in many adverse environmental consequences [3,4]. Waste water containing lignin that has been discharged from many industries (e.g., the paper industry, rice milling businesses, and traditional Chinese medicine businesses) has led to serious water pollution [5,6,7]. Agricultural wastes such as straw contain stable lignin, which limits the further application of cellulose and hemicellulose [4]. These biomass resources are often burned by farmers in China, which causes serious air pollution (SO_2_, NO_2_, CO_2_, and CO) in autumn and winter and affects human health [8]. Lignin degradation can help solve the environmental problems caused by lignin pollutant emissions or incineration [9]. At the same time, its degradation can improve the pretreatment efficiency of lignocellulose biomass, which results in the effective separation of cellulose, hemicellulose, and lignin and a mild conversion of biomass to bioenergy as well as other valuable products [3,10].

Currently, the main lignin degradation technologies are chemically, physically, and biologically based. Energy consumption during the physical processes is normally high and the chemical processes may cause secondary pollution from potential mismanagement of the chemicals used. On the other hand, the biodegradation method that uses some fungi and bacteria that have evolved in nature to deconstruct lignin is environmentally friendly and has relatively lower energy consumption [11,12].

Despite the above-mentioned advantages, the current lignin biodegradation approach has several drawbacks. The most active microbes with respect to lignin biodegradation that have been identified to date are fungi, such as those belonging to the white-rot or brown-rot families, which have been widely reported to be capable of decomposing wood [13]. However, these microorganisms often lack the capability required for industrial applications, such as fast growth rates [14]. Bacteria grow faster than fungi, and they have been reported to be able to produce high value-added products from lignin [15]. However, the decomposition and utilization of lignin by bacteria is limited [12]. To overcome this, researchers have tried to use protoplast technology to construct an inter-kingdom fusant (the protoplasts of fungi and bacteria were prepared, and then the cells were fused) [16]. They successfully prepared fusants of *Enterobacter cloacae* and *Psathyrella candolleana* to degrade alkali lignin [16]. However, the degradation efficiency of the fusants was sensitive to the environment, and their genetic stability was also not clear. In addition to living microorganisms, many enzymes involved in lignin degradation have been identified, such as laccase, lignin peroxidase (LiP), manganese peroxidase (MnP), dye-decolorizing peroxidase (DyP), and aryl-alcohol oxidase (AAO) [17,18]. Their mixtures have been used to degrade lignin [19] but with relatively high costs and nonuniversal adaptability for different substrates [20].

The decomposition of lignin in nature is a result of cooperation among fungi and bacteria in natural microbial consortia [21]. At present, more attention has been given to single lignin-degrading strains rather than to microbial consortia. Some studies have shown that microbial consortia have higher lignin degradation efficiencies than single strains [22,23] and that their ability to adapt to different environments is greater. For example, when treating different kinds of wastewater, the treatment efficiency of a microbial consortium is much higher than that of a single microorganism [24]. Given the potential advantages of microbial consortia, efficient lignin degradation by microbial consortia could be obtained by screening water-preserved wooden antiques from ancient graves [25]. As China has a long history and culture, large numbers of wooden antiques from various dynasties are unearthed each year. The hypotheses of our study are that carbohydrates such as cellulose and hemicellulose were consumed by microorganisms during long-term burial and that the remaining lignin created a good lignin-rich environment (>60% lignin content) for lignin-degrading microorganisms that could utilize lignin as a carbon source [26]. After these wood antiques were unearthed, they were soaked in water for further preservation to avoid dry shrinkage and deformation. During the process of burial and preservation after excavation, a continuous water-saturated state is helpful for microorganism survival, while the water-preserved wooden antiques are further decomposed [27]. Thus, it is feasible to obtain the lignin-degrading microbial consortia from samples of water-saturated preserved wood antiques.

In this study, several lignin-degrading microbial consortia were screened that were obtained from different wooden antique samples. Their degradation characteristics, consortium structures, and lignin degradation products were analyzed and compared. The findings from this research support further investigations of which consortia are best for use in biomass treatment for biofuel and biogas production, in the pulping process, and in treating lignin-rich wastewater from the paper industry, compositing leachates, rice milling sites [7], and traditional Chinese medicine businesses.

## 2. Results

### 2.1. Screening of Lignin-Degrading Microbial Consortia

**Figure 1:**
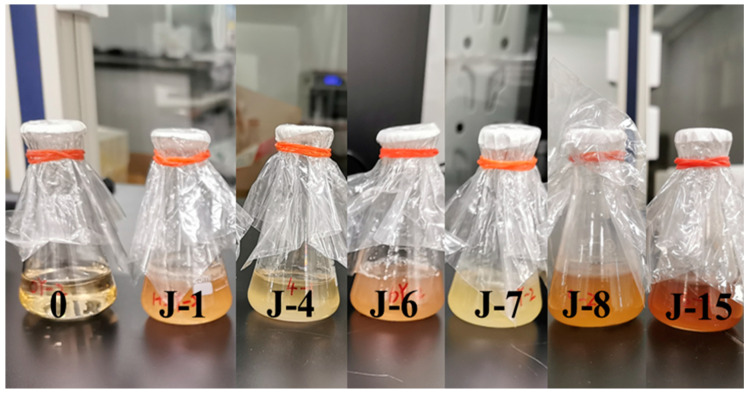
Color changes of different microbial consortiums in the guaiacol screening experiment.

The wooden antiques were preserved in water. Solid samples were obtained from pieces that fell off during the soaking process, and liquid samples were obtained from the soaking water. The typical color changes of microbial consortia in the guaiacol screening experiment are shown in Figure 1. According to the guaiacol color test, the lignin degradation efficiency by rotation incubation should be higher than that by static incubation. It was also observed that the pH of the basic culture medium increased during microbial consortium incubation. Among 40 samples, four microbial consortia, namely, J-1, J-6, J-8 and J-15, with the best performance were obtained after screening.

### 2.2. Comparative Study of the Lignin Degradation Performance of Different Microbial Consortia

To study the lignin degradation effects of the four selected microbial consortia under different conditions, their lignin degradation performances were studied. The control was defined as the same operational mode without adding microorganisms. The results showed that the lignin concentrations remained unchanged in the control.

**Figure 2:**
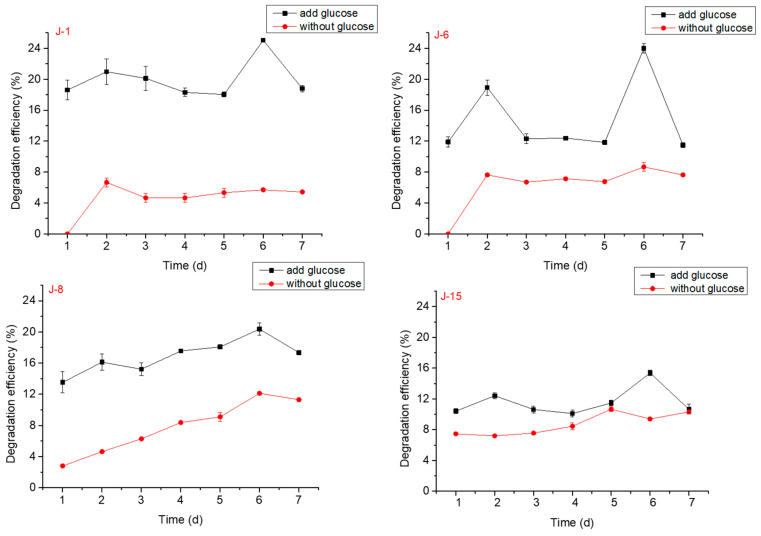
Effect of glucose on lignin degradation by J-1, J-6, J-8, and J-15 (pH = 6, rotation speed = 160 rpm; initial lignin concentration = 0.5 g/L).

The effect of glucose on lignin degradation by different microbial consortia was studied. The results showed that lignin with added glucose greatly improved the degradation efficiency when compared with lignin alone and reached higher efficiencies on the 1st, 2nd, and 6th days (Figure 2). In the follow-up experiments, glucose degradation system was still selected.

**Figure 3:**
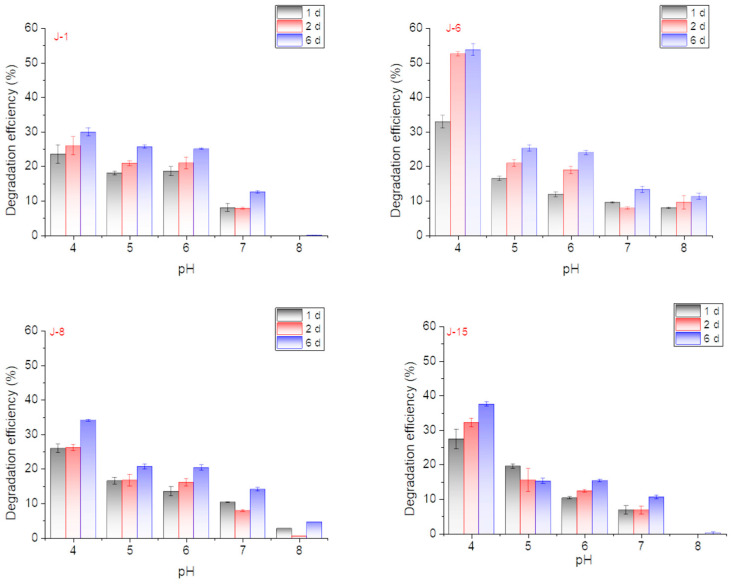
Effect of initial pH on lignin degradation efficiency by J-1, J-6, J-8, and J-15 (rotation speed = 160 rpm, initial lignin concentration = 0.5 g/L).

**Figure 4:**
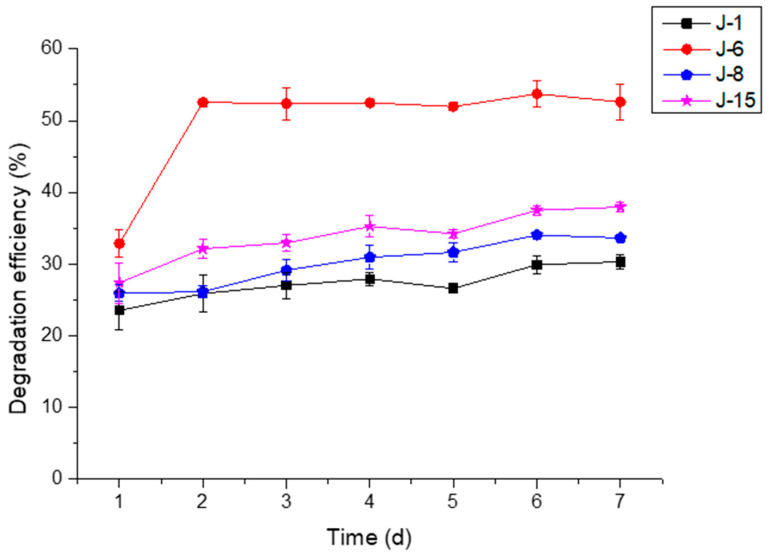
Effect of treatment time on lignin degradation by J-1, J-6, J-8, and J-15 (pH = 4, rotation speed = 160 rpm, initial lignin concentration = 0.5 g/L).

The effect of initial pH on lignin degradation by different microbial consortia was studied. The lignin degradation performance was best when the initial pH of the culture medium was 4 (Figure 3). Under conditions with a pH value = 4, the lignin degradation efficiency of J-6 reached 52.6% after 48 h and 53.7% on the 6th day. The degradation rates of the other microbial consortia reached 30% (J-1), 34% (J-8), and 37.5% (J-15) on the 6th day. Degradation experiments under optimal pH conditions also further confirmed that the degradation efficiencies on the 1st and 2nd days were often higher and then reached a plateau on the 6th day (Figure 4).

**Figure 5:**
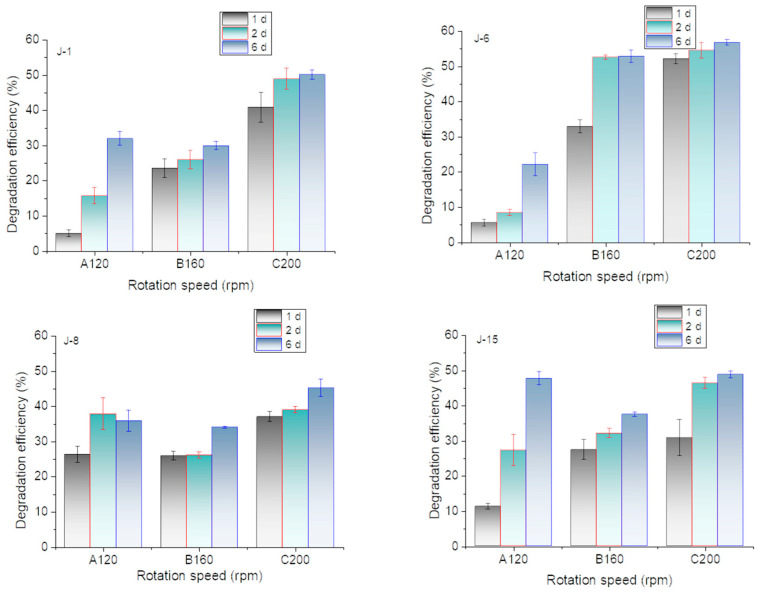
Effect of rotation speed on lignin degradation efficiency by J-1, J-6, J-8, and J-15 (pH = 4, initial lignin concentration = 0.5 g/L).

Previous experiments have shown that microorganisms have a certain demand for oxygen in the processes of growth and lignin utilization because higher degradation efficiencies were achieved by rotation incubation. In this research, the effect of rotation speed on lignin degradation is shown in Figure 5. The results showed that the lignin degradation efficiencies of all microbial consortia reached 45% at 200 rpm on the 6th day. However, J-8 and J-15 also exhibited good performances at low rotation speeds.

**Figure 6:**
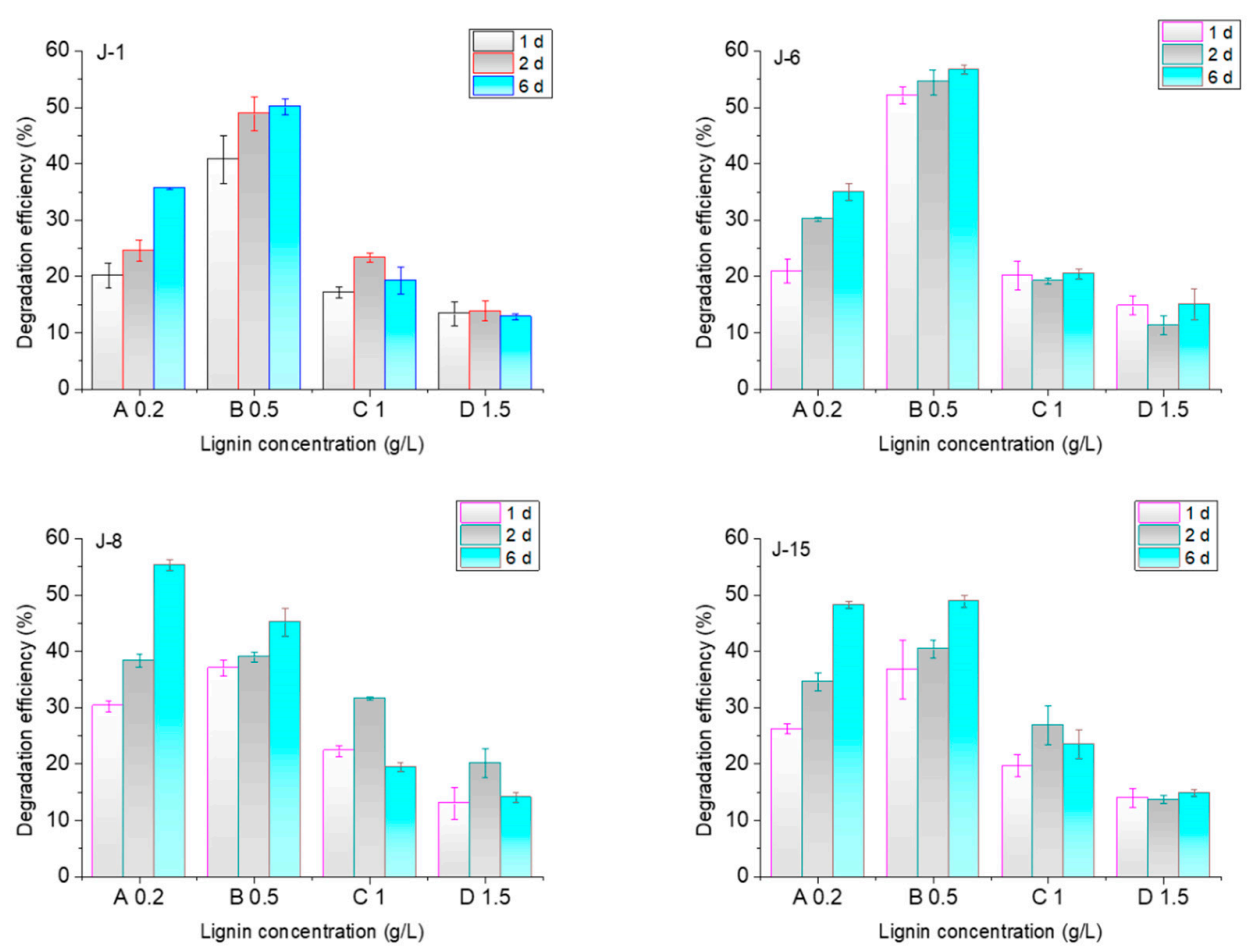
Effect of initial lignin concentration on lignin degradation efficiency by J-1, J-6, J-8, and J-15 (pH = 4, rotation speed = 200 rpm).

The effects of lignin concentrations on the lignin degradation efficiencies of the four microbial consortia are shown in Figure 6. In the initial experiments, the lignin concentration was 0.5 g/L. When lignin concentrations were higher than 0.5 g/L, the lignin degradation efficiencies of the four microbial consortia decreased. When lignin concentrations were lower than 0.5 g/L, the lignin degradation efficiency of J-8 improved on the 6th day, while those of J-1 and J-6 decreased significantly and that of J-15 decreased slightly. According to one-way ANOVA with Tukey’s test, the lignin concentration had a significant impact on the degradation efficiency of J-1 and J-6 but not on J-8 and J-15.

At the same time, degradation experiments at different temperatures were also carried out. The results showed that temperature had a negligible effect on lignin degradation within a temperature range of 25–35 °C.

**Figure 7:**
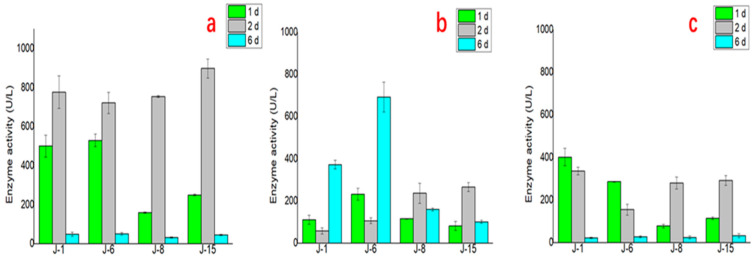
The lignin degradation enzyme activity test of the four microbial consortiums at different times ((**a**): laccase; (**b**): LiP; (**c**): MnP).

Furthermore, the results of lignin degradation enzyme activity tests of the four microbial consortia at different times are shown in Figure 7. The enzyme activity changes of the three important enzymes in the process of lignin degradation differed. For laccase, the enzyme activities of the four microbial consortia J-1, J-6, J-8, and J-15 were higher on the 1st and 2nd days and decreased significantly on the 6th day. For lignin peroxidase (LiP), J-1 and J-6 reached the highest enzyme activity on the 6th day. For manganese peroxidase (MnP), the activity was higher on the 1st day and 2nd day but decreased significantly on the 6th day.

### 2.3. Comparative Study of the Consortium Composition

#### 2.3.1. Comparison of the Fungal Consortium Composition

**Figure 8:**
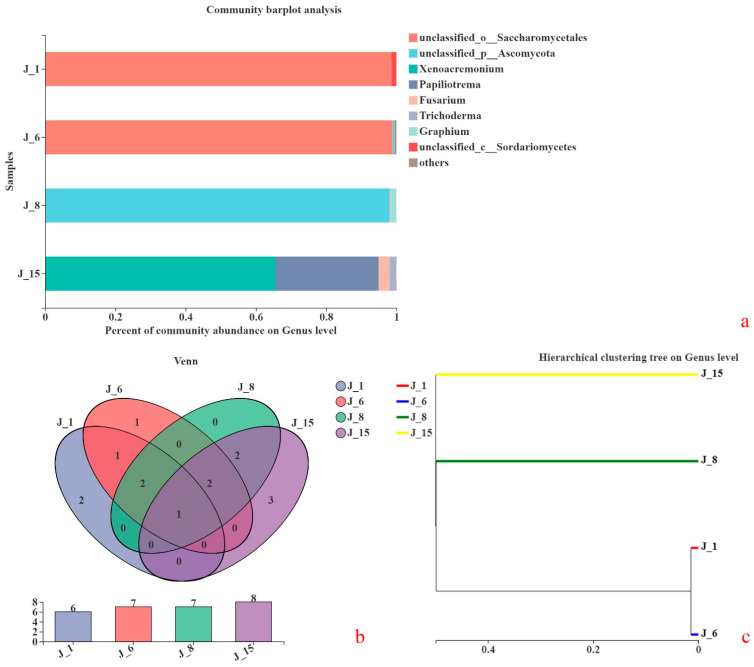
Comparison of fungal consortium composition ((**a**): consortium structure; (**b**): Venn diagram; (**c**): cluster analyses).

According to genomic sequencing analyses, the consortium compositions of the four groups are shown in Figure 8a. At the genus level, the fungi of J-1 were composed of Saccharomycetales 98.56% and Sordariomycetes 1.43%. The fungi of J-6 were composed of Saccharomycetales 98.92% and Ascomycota 0.56%. The fungi of J-8 included Ascomycota 98.15% and *Graphium* sp. 1.63%. The fungi of J-15 included *Xenoacremonium* sp. 65.61%, *Papiliotrema* sp. 29.33%, *Fusarium* sp. 3.08%, and *Trichoderma* sp. 1.93%.

A Venn diagram was used to count the fungal compositions of the four groups, as shown in Figure 8b. In general, Operational Taxonomic Units (OTUs) with a similarity level of 97% were selected for analysis. A Venn diagram was used to calculate the number of common and unique species in multiple groups or samples. *Mortierella* sp. was found in J-1, J-6, J-8, and J-15 based on the sequencing results.

To study the similarities or differences among different sample consortium structures, cluster analysis was carried out on the sample consortium distance matrix, and a sample hierarchical clustering tree was also constructed (Figure 8c). The UPGMA (unweighted pair group method with arithmetical mean) algorithm was used to construct a tree structure to visually represent the degrees of similarity or difference in the consortium compositions of different environmental samples. The consortium compositions of J-1 and J-6 were very close but were quite different from those of J-8 and J-15. Differences between J-8 and J-15 were also obvious.

**Figure 9:**
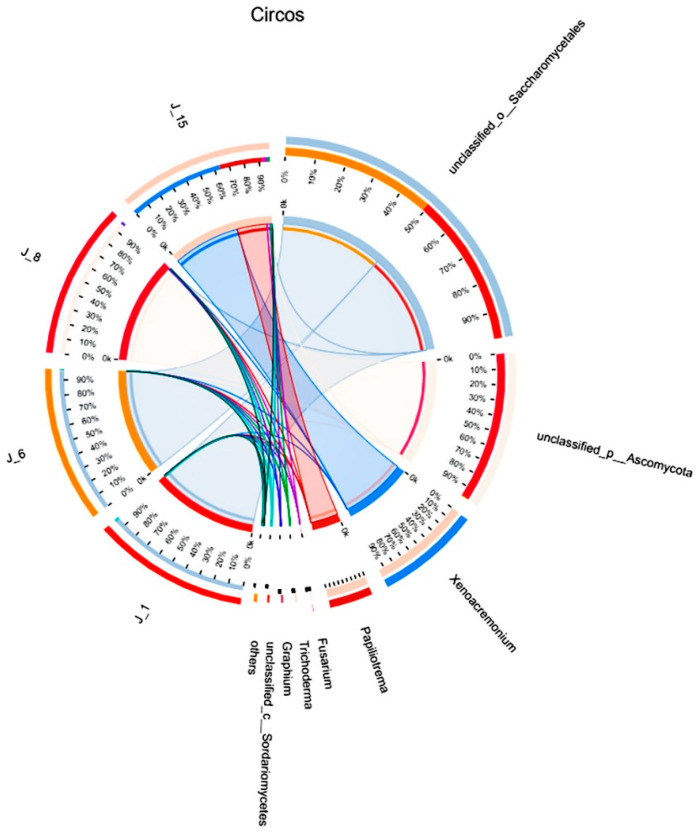
Circos sample and species relationship diagram.

According to the Circos sample and species relationship diagram (Figure 9), Saccharomycetales in J-1 and J-6 accounted for 100% of the total amount of Saccharomycetales in the four groups. Ascomycota in J-8 accounted for 99% of the total Ascomycota in the four groups, and *Fusarium* sp. and *Trichoderma* sp. in J-15 accounted for the majority (more than 90%).

**Figure 10:**
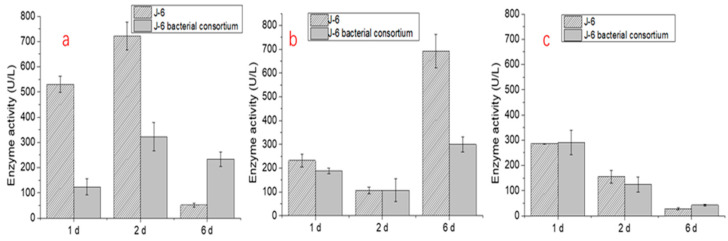
Lignin degradation enzyme activity of the original J-6 microbial consortium and J-6 bacterial consortium ((**a**): laccase; (**b**): LiP; (**c**): MnP).

To determine the effect of yeast on the activity of lignin-degrading enzymes, J-6 cells were treated with nystatin to inhibit yeast activity. Then, the J-6 bacterial consortium was cultured and transferred to a lignin degradation medium to compare the changes in lignin degradation enzyme activity. The results (Figure 10) showed that, when compared with the original J-6 microbial consortium, the activities of lignin-degrading enzymes, especially laccase and LiP, decreased significantly in the degradation medium of the J-6 bacterial consortium.

#### 2.3.2. Comparison of the Bacterial Consortium Composition

**Figure 11:**
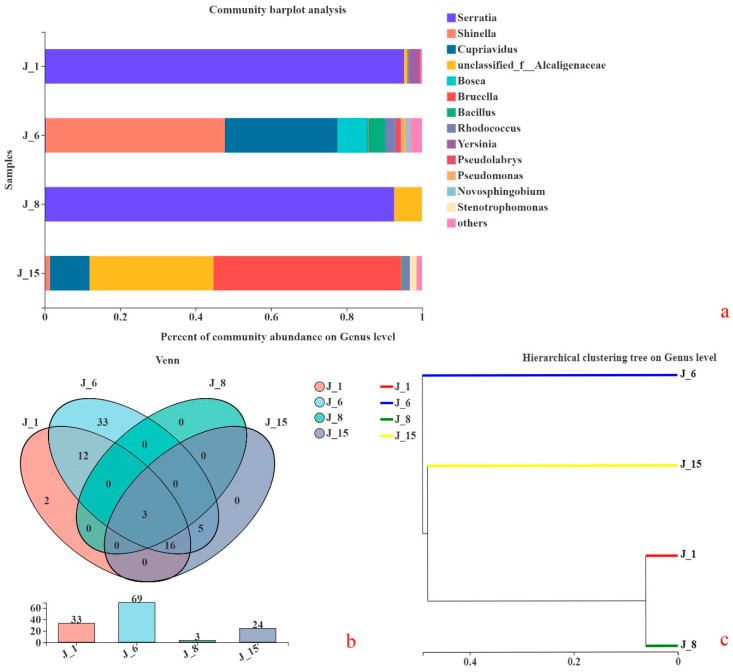
Comparison of bacterial consortium composition ((**a**): consortium structure; (**b**): Venn diagram; (**c**): cluster analyses).

The bacterial compositions of the four microbial consortia were analyzed, and their consortium structures are shown in Figure 11a. The bacterial composition of J-1 was as follows: *Serratia* sp. 95.23% and *Yersinia* sp. 2.33%. The bacterial composition of J-6 was *Shinella* sp. 47.38%, *Cupriavidus* sp. 29.84%, *Bosea* sp. 7.96%, and *Bacillus* sp. 4.39%. The bacterial composition of J-8 was *Serratia* 92.59%, Alcaligenaceae 7.40% and *Cupriavidus* sp. 0.1%. The bacterial composition of J-15 was Alcaligenaceae 32.85%, *Brucella* sp. 49.74%, *Cupriavidus* sp. 10.49%, and *Rhodococcus* sp. 1.92%.

A Venn diagram was used to count the bacterial compositions of the four groups, as shown in Figure 11b. The common species of J-1, J-6, J-8, and J-15 were Alcaligenaceae, *Cupriavidus* sp. and *Serratia* sp.

In addition, cluster analyses were carried out on the sample consortium distance matrix to construct a sample hierarchical clustering tree. The results are shown in Figure 11c. J-1 and J-8 show very similar bacterial composition, whereas both J-6 and J-15 are different in their composition.

**Figure 12:**
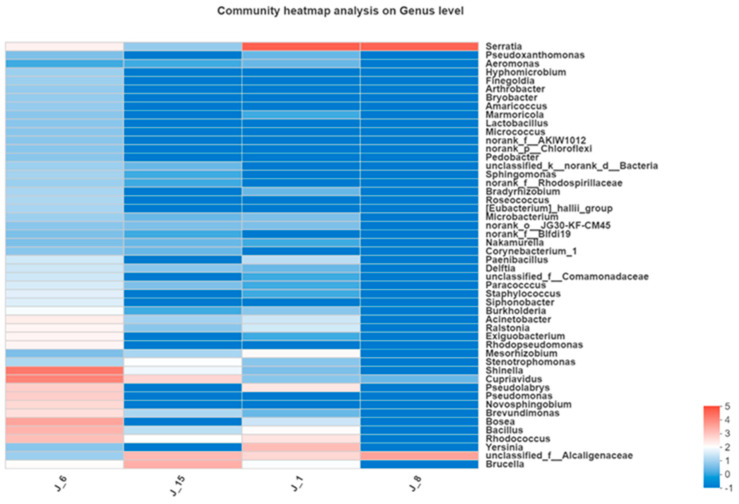
Heatmap of bacterial composition.

A color gradient was utilized in a heatmap to represent the information for species compositions and abundances (Figure 12). J-6 had the highest abundance, while J-8 had the lowest bacterial abundance. J-6 mainly included *Shinella* sp., *Cupriavidus* sp., *Bosea* sp., *Bacillus* sp., *Rhodococcus* sp., and *Pseudomonas* sp.

### 2.4. Comparison of the Biomass of Fungi and Bacteria in the Microbial Consortia

**Figure 13:**
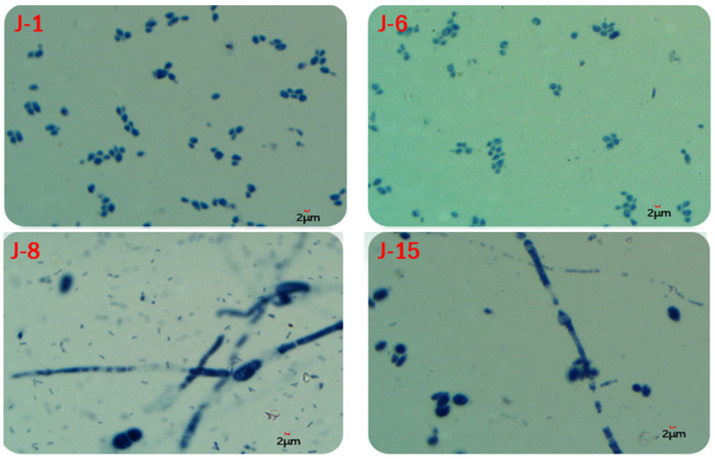
Microscopic examination of lignin degradation microbial consortiums.

The biomass results showed that the fungal biomass of J-8 and J-15 accounted for 66% and 84% of the total biomass, respectively, while those of J-1 and J-6 accounted for 40% and 31% of the total biomass, respectively. In addition, microscopic examination (Figure 13) showed that fungal hyphae developed in J-8 and J-15, while J-1 and J-6 contained oval and spherical single cells, and no large fungal hyphae were present.

### 2.5. Comparison of the Lignin Degradation Products

The degradation products of each degradation group were further studied. Samples were obtained after the 1st, 2nd, and 6th days of degradation. The results are shown in Table 1. Each microbial consortium formed different degradation products. The types of products studied included not only the final products but also some intermediate products of the metabolic processes. For example, the specific products of J-1 included 2-[2-[3,4,5-trimethoxyphenyl] ethenyl]-5,6,7,8-tetramethoxy-3-methyl-chromone. The specific products of J-6 included 2-methoxy-4-vinylphenol and benzenebutyric acid, 2,3-dimethoxy-. The specific products of J-8 included 3-hydroxybenzoic acid and 5,5′-dimethoxy-3,3′, 7,7′-tetramethyl-2,2′-binaphthalene-1,1′,4,4′-tetrone. The specific products of J-15 included 1,2-benzenedicarboxylic acid and butyl octyl ester. Lignin degradation by J-6 was relatively complete, and there were no degradation products that contained multiple benzene rings.

## 3. Discussion

According to the guaiacol color test, the lignin degradation efficiency by rotation incubation should be higher than that by static incubation. This may indicate that the selected lignin-degrading microorganisms have a certain oxygen demand during the degradation process, which was similar to the conclusion of other researchers [9,28]. At the same time, simple mechanical movement may bring better mixing of matrix and microorganism and dissolving of medium. It was also observed that the pH of the basic culture medium increased during microbial consortium incubation, which was similar to findings reported in the literature [29].

It was reported that some microorganisms could break down lignin, but it was necessary to add glucose as an energy source [30]. In view of previous research, we also determined the degradation rates after adding glucose in the degradation experiments. Similar to these studies, in this research, microorganisms exhibited strong adaptability to glucose. The results showed that lignin with added glucose greatly improved the degradation efficiency when compared with lignin alone. These results echo the findings from other work that glucose addition may effectively improve lignin degradation by promoting the growth of microorganisms and secreting the ligninolytic enzymes [31].

In a lignin degradation system, the pH value has been reported to be an important factor [29]. pH is presumed to have certain impacts on lignin degradation, whether for degradation by a single microorganism or by microbial consortia. According to the literature, for most lignin-degrading systems, neutral conditions were better. For example, the optimal pH value of *Aneuribacillus aneurilyticus* was 7.6 [32] and that of *Comasonas* sp. B-9 was 7 [33]. DM-1, which is a lignin degradation microbial consortium reported in the literature, also preferred neutral conditions [29]. However, in this study, the lignin degradation performance was best when the initial pH of the culture medium was 4. Better degradation under acidic conditions may also correspond to the microorganism consortium composition and enzyme activities of lignin-degrading enzymes. It was reported that under acidic conditions, the strain could produce more lignin-degrading enzymes, such as laccase, with high enzyme activity [34,35].

Many literature results have shown that rotation speed of incubation had a significant impact on the microbial degradation process of aromatic compounds and lignin. For example, ferulic acid degradation by *Phomopsis liquidambari* was carried out under shaking incubation conditions at an optimal speed of 180 rpm [36]. Bacillus subtilis was used in the pretreatment process of corn stalks to remove lignin and improve the production capacity of subsequent products, during which the optimal rotation speed was 120 rpm [15]. *Pandoraea* sp. B-6 degraded lignin and reached the highest degradation efficiency at 120 rpm [37]. In this research, the results showed that the lignin degradation efficiencies of all microbial consortia reached 45% at 200 rpm on the 6th day. The increase of rotation speed is beneficial to the growth of aerobic microorganisms [35]. Aerobic microorganisms played a greater role in the lignin degradation. Taking J-6 as an example, there were a large number of yeasts and aerobic bacteria in J-6. Oxygen supply can significantly enhance the growth of microorganisms and ultimately improve the degradation of lignin.

Lignin concentration is an important factor that affects degradation efficiency [7]. Due to the different compositions of the four groups, the effect of lignin concentration on degradation by J-1, J-6, J-8, and J-15 was quite different. The main fungi in J-1 and J-6 was yeast, and yeast growth was greatly affected by lignin concentration, according to our former study. Therefore, lignin concentration also has a significant effect on lignin degradation by J-1 and J-6. An advantage of microbial consortia is that they have the ability to adapt to different environments, including various lignin concentrations. However, domestication is important and required prior to lignin degradation experiments [38].

At the same time, degradation experiments at different temperatures showed that microbial communities had strong practical applications in a temperature range close to room temperature.

In summary, the main factors that affected lignin degradation efficiency were pH, lignin concentration, and rotation speed. Under optimal conditions, the degradation efficiencies of the four microbial consortia in this study were above 45% after 6 days of incubation. The degradation efficiency of the J-6 microbial consortium reached 54% after 48 h with an initial lignin concentration of 0.5 g/L at an initial pH value of 4 and rotation speed of 200 rpm. To the best of our knowledge, this study also demonstrated the superiority of the degradation ability of microbial consortia over other reported microorganisms (Table 2).

Furthermore, to explain the higher lignin degradation efficiency of microbial consortia, the lignin degradation enzyme activity of the four microbial consortia was compared. Regarding the test results for the corresponding enzyme activities of the characteristics of lignin degradation of the four communities, the enzyme activities of J-1 and J-6 were closer, which may be related to the similar composition of J-1 and J-6. J-6 had similar laccase and MnP activities but higher LiP enzyme activity than the other microbial consortia, which explains why it had the highest lignin degradation efficiency. In this study, the highest laccase activity of the various microbial consortia reached 897.5 ± 48.5 U/L (J-15 on the 2nd day), and the MnP activity reached 401.6 ± 41.1 U/L (J-1 on the 1st day). Meanwhile, compared with the performance of some bacteria, LiP was not produced in the degradation process [37]. The microbial consortia used in this study can produce three kinds of enzymes at the same time, with the highest activity of LiP reaching 691.9 ± 71.1 U/L (J-6 on the 6th day). These results also proved that lignin degradation by microbial consortia was superior to that by a single microorganism.

The compositions of lignin-degrading microbial consortia correspond to their lignin degradation characteristics and performances [42]. Through the analysis of fungal composition, it was shown that *Mortierella* sp. was found in J-1, J-6, J-8, and J-15. This microorganism may degrade lignin or lignin-like structural substances. Gong and coworkers [43] also showed that *Mortierella* sp. has a higher degradation ability and faster degradation rate for low-ring PAHs (polycyclic aromatic hydrocarbons). The degradation ability of mixed microorganisms that were composed of *Mortierella* sp. and *Pseudomonas* sp. was better than that of a single microorganism. Meanwhile, the results show that a mixed consortium of fungi and bacteria was better than a single microorganism for degrading polymer compounds with benzene rings. The UPGMA algorithm was used to construct a tree structure to visually represent the degrees of similarity or difference in the consortium compositions of different samples. The difference in fungal consortium compositions of J-1 and J-6 was the smallest. The lignin degradation characteristics of J-1 and J-6 were also very similar.

Saccharomycetales in J-1 and J-6 accounted for 100% of the total amount of Saccharomycetales in the four groups. *Fusarium* sp. and *Trichoderma* sp. in J-15 accounted for the majority (more than 90%). *Fusarium* sp. [44,45] and *Trichoderma* sp. [46] were reported in the literature with respect to lignin-degrading fungi but less was reported regarding Saccharomycetales. However, the lignin degradation efficiency of J-6 was relatively high, which indicated that the Saccharomycetales microorganism can survive in the lignin substrate media and may have promoted lignin degradation. In this study, glucose was added to the lignin degradation system, which promoted the reproduction of Saccharomycetales. At the same time, the culture conditions were acidic, which corresponded to the characteristics of yeast growth. To determine the effect of yeast on the activity of lignin-degrading enzymes, J-6 was treated with nystatin to inhibit yeast activity. The results indicated that yeast in the microbial consortium improved the enzyme activity of lignin-degrading enzymes and thus increased the lignin degradation efficiency. There are also similar reports in the literature; for example, Šlosarčíková et al. [47] reported that although yeast did not have the ability to degrade dye chemicals, it could survive in a biphenyl dye solution, and in a degradation system with yeast, the activity of lignin-degrading enzymes such as laccase was improved. In addition, it has been reported that yeast can produce lignin-degrading enzymes in the degradation system of benzene compounds [48]. Furthermore, the rapid growth of yeast and its consumption of glucose also promoted lignin utilization by other microorganisms in the consortium.

Through the analysis of bacterial composition, it was shown that Alcaligenaceae, *Cupriavidus* sp. and Serratia sp. were found in J-1, J-6, J-8, and J-15. These three microorganisms have also been reported in other literature. For example, a lignin-degrading consortium was obtained from a sugarcane plantation soil sample, and taxonomic analyses (based on 16S rRNA) indicated the prevalence of Proteobacteria, Actinobacteria, and Firmicutes members, including the Alcaligenaceae and Micrococcaceae families, which were enriched in lignin-degrading consortia compared to sugarcane soil [49]. Kraft lignin was able to be used as the sole carbon source of *Cupriavidus basilensis* B-8, and 41.5% of the lignin, 37.7% of the total carbon (TC), and 43.0% of the color were removed after 7 days of incubation [50]. *Serratia* sp. that was isolated from effluent-contaminated soil was able to degrade and detoxify pulp and paper mill effluent with significant lignin reductions [51]. Further studies to identify the common microorganisms in different lignin-degrading consortia can provide insights into obtaining lignin-degrading bacteria. J-6 had the highest abundance, while J-8 had the lowest bacterial abundance. J-6 mainly included *Shinella* sp., *Cupriavidus* sp., *Bosea* sp., *Bacillus* sp., *Rhodococcus* sp., and *Pseudomonas* sp. These types of microorganisms have some applications in the field of lignin degradation. For example, laccase genes in the genera *Shinella* sp. were detected, which indicated that its metabolic activity was related to lignin degradation [52]. *Bosea* sp. and *Bacillus* sp. can also produce laccase enzyme for lignin degradation [53]. Some bacteria belonging to *Rhodococcus* sp. have been determined to have the capability to degrade lignin, and among various Rhodococcus species, *R. jostii* RHA1 is the one that has been studied most often [13]. A bacterial peroxidase was purified from *Pseudomonas* sp. SUK1, and this 86-kDa heme-containing peroxidase showed optimal activity on a range of lignin-related phenols [53]. In summary, according to our lignin degradation performance results and consortium taxonomic analyses, the reason for the high degradation efficiency of J-6 was the presence of abundant bacterial communities.

All four microbial consortia had certain lignin degradation abilities. Lignin degradation may be carried out by fungi or bacteria, and through the synergies between fungi and bacteria, the degradation ability of lignin was improved. Combined with results of our degradation experiments, the impact of rotation speed and lignin concentration on bacteria was more obvious, while that on fungal lignin degradation was smaller.

According to other literature [5,54,55], the types of lignin degradation products included benzene acetic acid compounds, benzaldehyde compounds, cinnamic acid compounds, phenol-based compounds, benzoic acid compounds, benzyl benzoate compounds, phthalate compounds, benzyl alcohol compounds, veratryl alcohol compounds, guaiacol compounds, vanillin compounds, acetophenone compounds, and phenyl acetate compounds. In this study, the classes of products that resulted from digestion by the four microbial consortia included benzaldehyde compounds, benzoic acid compounds, phenol, benzene acetic acid compounds, acetophenone, and phthalate ester compounds, and the types of intermediate products that were produced in the degradation process were also detected and reported, which provided a basis for future research on lignin degradation products. Hexadecanoic acid and octadecanoic acid, which were products obtained in this study, were also detected in the GC analysis of lignin degradation by other microorganisms [4]. Lignin degradation by J-6 was relatively complete, which also indicated that lignin degradation by bacterial communities was more complete than that by fungal communities [21].

## 4. Materials and Methods

### 4.1. Materials

The samples for lignin degradation microbial consortium screening were collected from the Jingzhou Museum of Hubei Province (30°21′20″ N, 112°10′30″ E). The wooden antiques consisted mainly of wooden components, chime frames, wooden wares, and coffins that were unearthed from the No. M190 noble tomb of Zeng State (the middle of the Spring and Autumn period from approximately 2600 years ago) in Zaoshulin, Suizhou, Hubei Province. Wood chips that were scattered on the surface of the antiques and their soaking water samples were collected in sterile sampling bags/tubes, sealed and frozen for transportation. Then, these samples were stored at 4 °C in a refrigerator. The alkali lignin used in the experiment was purchased from Sigma with a molecular weight of 10,000 (CAS:8068-05-1). Other reagents (analytical grade) were purchased from the Beijing Chemical Factory.

### 4.2. Culture Conditions

The primary screening medium contained peptone 2.5 g/L, KH_2_PO_4_ 1 g/L, NaCl 2.5 g/L, MgSO_4_·7H_2_O 0.2 g/L, CaCl_2_ 0.1 g/L, guaiacol 1 mL/L and trace elements l mL/L. The basic culture medium for screening contained peptone 2.5 g/L, KH_2_PO_4_ 1 g/L, NaCl 2.5 g/L, MgSO_4_·7H_2_O 0.2 g/L, CaCl_2_ 0.1 g/L, different carbon sources and trace elements l mL/L. The trace element solution included ZnSO_4_·7H_2_O 0.1 g/L, CoCl_2_·6H_2_O 0.16 g/L, CuSO_4_·5H_2_O 0.15 g/L, MnSO_4_·H_2_O 0.1 g/L, H_3_BO_3_ 0.02 g/L, Na_2_MoO_4_·2H_2_O 0.8 g/L, and NiCl_2_·6H_2_O 0.05 g/L, and the pH was the original value, which was 6.08 [29]. The medium for the lignin degradation test contained ammonium sulfate 2.5 g/L, KH_2_PO_4_ 1 g/L, NaCl 2.5 g/L, MgSO_4_·7H_2_O 0.2 g/L, CaCl_2_ 0.1 g/L, glucose 0.5 g/L, trace element l mL/L and alkali lignin of different concentrations.

### 4.3. Screening of Microbial Consortia

Samples (1 g) were added to sterilized normal saline (9 mL), stirred for 30 min, and were then allowed to stand for 10 min. Then, 2 mL of supernatant was selected and added to 18 mL of primary screening medium and incubated at 30 °C for 7 d without shaking. Another 2 mL of supernatant was transferred to the basic medium (18 mL), which also contained guaiacol at a concentration of 1 mL/L, and cultured at 120 rpm and 30 °C for 7 d. The purpose of static culture and shock culture was to test the effect of oxygen on microbial consortium growth. After 7 days, the medium color was observed. The red and pink media after static incubation and rotation incubation were selected. Five milliliters of microbial suspension were added to 45 mL of basic culture medium containing alkali lignin at a concentration of 1 g/L for the second screening, which was followed by static or rotation (160 rpm) incubation at 30 °C for 7 days. The microbial suspension obtained from the second screening was transferred to a lignin degradation medium with a lignin concentration of 0.5 g/L and 10% inoculum volume. The microbial were cultured at pH 7, 30 °C, and 160 rpm for 7 d. The microbial consortia with relatively high lignin degradation efficiencies were selected for the lignin degradation experiments.

### 4.4. Lignin Degradation Experiments

Three cycles of incubation in the lignin degradation medium were carried out prior to the lignin degradation experiments. Consortium composition analyses of the lignin degradation microbial consortia after stable culture were carried out. The lignin degradation experiment was carried out by changing the culture conditions. The control was set as the same operation mode without adding microorganisms. Culture conditions, including the carbon source (“lignin alone” and “lignin plus glucose”), pH (4–8), rotation speed (120 rpm, 160 rpm and 200 rpm; on a shaking incubator with a rotational radius of 10 cm), and lignin concentration (0.2–2 g/L), were changed to obtain the optimal degradation efficiency.

### 4.5. Analytical Methods

Microbial consortium genomic DNA was extracted from the samples using the E.Z.N.A.^®^ soil DNA Kit (Omega Bio-Tek, Norcross, GA, USA). The bacterial 16S rRNA gene was amplified with the primer pair 515F (5′-GTGCCAGCMGCCGCGG-3′) and 806R (5′-GGACTACHVGGGTWTCTAAT-3′). The fungal ITS gene was amplified with the primer pair ITS1F (5′-CTTGGTCATTTAGAGGAAGTAA-3′) and ITS2R (5′-GCTGCGTTCTTCATCGATGC-3′) by an ABI GeneAmp^®^ 9700 PCR thermocycler (ABI, CA, California, USA). The purified amplicons were sequenced on an Illumina MiSeq PE300 platform/NovaSeq PE250 platform (Illumina, San Diego, CA, USA). The raw reads were deposited into the NCBI Sequence Read Archive (SRA) database (Accession Number: PRJNA666498). The sequence data processing method was carried out according to the literature [56,57,58]. Enzyme activities for lignin degradation (laccase, LiP, and MnP) were determined according to the literature [37]. Laccase activity was measured by monitoring the oxidation of ABTS. Lip activity was measured by monitoring the oxidation of veratryl alcohol. MnP activity was measured by monitoring the oxidation of 2, 6-DMP. When measuring the enzyme activity of the J-6 bacterial consortium, 0.1 g/L nystatin was directly added to the J-6 consortium. After a shaking treatment for 1 h, the cells were transferred to a culture medium that contained 0.1 g/L nystatin and were cultured for 12 h. The cells were then transferred to a nystatin medium and were cultured for 12 h again to obtain a J-6 bacterial consortium.

The lignin concentration was measured by measuring the absorbance at 280 nm according to a previously reported method [59]. The lignin degradation efficiency was calculated as follows:

Degradation efficiency (%) = (initial lignin concentration − lignin concentration after treatment)/initial lignin concentration.

GC-MS analysis of the lignin degradation products was conducted according to the reference [37]. Fungal and bacterial biomasses were measured by a classic method called the respiratory inhibition method [60]. Agar slices were used for microscopic examination of microbial consortia, and samples were placed in a microscope (cx23, Olympus, Japan) for microscopic examination.

### 4.6. Statistical Methods

All of the above experiments were repeated in triplicate, and the average data were reported. One-way ANOVA with Tukey’s test was used to detect any significant differences among treatments (*p* < 0.05).

## 5. Conclusions

Interest in biological lignin degradation has been widely presented in the field of biomass utilization and wastewater treatment containing lignin compounds. In this study, we used antique wood samples as a novel, to the best of our knowledge, source for screening lignin degradation microbial consortia. Four microbial consortia, namely, J-1, J-6, J-8, and J-15, with lignin degradation capabilities were obtained, among which the J-6 microbial consortium was the most efficient, as was supported by the appearance of three lignin degradation enzymes with higher activities. The degradation efficiency of the J-6 microbial consortium reached 54% after 48 h with an initial lignin concentration of 0.5 g/L at an initial pH value of 4 and rotation speed of 200 rpm. Lignin degradation by J-6 was relatively complete, and there were no degradation products containing multiple benzene rings. The fungal consortium of J-6 was mainly composed of Saccharomycetales 98.92% and Ascomycota 0.56%, which accounted for 31% of the total biomass. Saccharomycetales adapted to the lignin substrate and promoted lignin degradation. Yeast in the microbial consortium improved the enzyme activity of lignin-degrading enzymes and thus increased the lignin degradation efficiency. The main bacteria in J-6 were *Shinella* sp. 47.38%, *Cuprividus* sp. 29.84%, and *Bosea* sp. 7.96%. The abundance of the bacterial consortium in J-6 may also be beneficial for lignin degradation. The J-6 microbial consortium demonstrated promising potential for applications with lignin degradation/removal purposes.

## Figures and Tables

**Table 1:** Identification of the metabolic products.

No.	RT ^1^	Compound	J-1			J-6			J-8			J-15		
			1d	2d	6d	1d	2d	6d	1d	2d	6d	1d	2d	6d
1	10.45 min	Phenol						+						
2	11.58 min	Ethyl 3-hydroxybutyrate				+	+					+		+
3	13.27 min	Benzaldehyde, 3-methyl	+	+	+	+	+	+	+	+	+	+	+	+
4	13.91 min	Salicylic acid			+									
5	13.92 min	3-Hydroxybenzoic acid							+					
6	14.27 min	2,6-Dihydroxyacetophenone	+											
7	14.61 min	2,6-Dihydroxybenzoic acid	+						+			+		
8	14.84 min	2-Ethylhexanoic acid			+			+			+			+
9	16.81 min	Benzeneacetic acid, 3-pentadecyl ester					+							
10	17.19 min	2-Methoxy-4-vinylphenol						+						
11	18.32 min	5,5′-Dimethoxy-3,3′,7,7′-tetramethyl-2,2′-binaphthalene-1,1′,4,4′-tetrone							+					
12	19.81 min	Butylated Hydroxytoluene			+	+	+	+	+	+	+	+	+	+
13	20.42 min	Tyrosol, acetate			+									
14	22.12 min	Cinnamic acid, 4-hydroxy-3-methoxy-, (5-hydroxy-2-hydroxymethyl-6-[2-(4-hydroxy-3-methoxyphenyl)ethoxy			+									
15	22.79 min	Benzenebutyric acid, 2,3-dimethoxy-				+								
16	23.77 min	1,2-Benzenedicarboxylic acid, butyl octyl ester												+
17	23.77 min	Phthalic acid, hept-3-yl isobutyl ester	+			+			+	+	+	+	+	
18	24.64 min	n-Hexadecanoic acid	+	+	+	+	+	+	+	+	+	+	+	+
19	24.71min	Dibutyl phthalate	+	+	+	+	+	+	+	+	+	+	+	+
20	26.51 min	Octadecanoic acid	+	+	+	+	+	+	+	+	+	+	+	+
21	30.14 min	Phenol, 2,4-bis(1-phenylethyl)-			+									+
22	31.79 min	Chromone,2-[2-[3,4,5-trimethoxyphenyl]ethenyl]-5,6,7,8-tetramethoxy-3-methyl-	+											
23	32.69 min	1-Hydroxy-2-(2,3,4,6-tetra-O-acetyl-beta-D-glucopyranosyl)-9H-xanthene-3,6,7-triyl triacetate	+		+						+	+	+	+

^1^ RT: retention time.

**Table 2:** Comparison of the degradation efficiency by J-6 and other microorganisms.

Microbial Strains	Lignin Degradation%		Degradation Time	Lignin Load
Bacteria				
*Bacillus flexus* [7]	20%		9 d	0.4 g/L
*Cupriavidus basilensis B-8* [37]	38%		7 d	0.5 g/L
*Citrobacter freundii* [9]	49%		6 d	600 ppm
Fungi				
*Cladosporium* sp. *Bio-1* [39]	35%		10 d	2.0 g/L
*Penicillium chrysogenum* [40]	83.50%		30 d	1.0 g/L
*Phellinus* sp. [41]	36%		10 d	0.50%
J-6	54%		48 h	0.5 g/L

## Data Availability

Most of the recorded data are available in all tables and figures in the manuscript and supplementary material.

## Sample Availability

Samples of the compounds are not available from the authors.
